# Exploring the K isotope composition of Göttingen minipig brain regions, and implications for Alzheimer's disease

**DOI:** 10.1093/mtomcs/mfac090

**Published:** 2022-11-23

**Authors:** Brandon Mahan, Theo Tacail, Jamie Lewis, Tim Elliott, Mette Habekost, Simon Turner, Roger Chung, Frédéric Moynier

**Affiliations:** IsoTropics Geochemistry Lab, Earth and Environmental Science, James Cook University, Townsville, Queensland 4814, Australia; Thermo Fisher Isotope Development Hub, Department of Earth and Planetary Sciences, Macquarie University, Sydney, New South Wales 2109, Australia; Department of Biomedical Research, Macquarie University, Sydney, New South Wales 2109, Australia; Bristol Isotope Group, School of Earth Sciences, University of Bristol, Bristol BS8 1RJ, UK; Institute of Geosciences, Johannes Gutenberg University, Mainz 55099, Germany; Bristol Isotope Group, School of Earth Sciences, University of Bristol, Bristol BS8 1RJ, UK; Bristol Isotope Group, School of Earth Sciences, University of Bristol, Bristol BS8 1RJ, UK; Department of Biomedicine, Aarhus University, 8000 Aarhus C, Denmark; Center for Neuroscience, University of Copenhagen Faculty of Health and Medical Sciences, 2200 Copenhagen N, Denmark; Thermo Fisher Isotope Development Hub, Department of Earth and Planetary Sciences, Macquarie University, Sydney, New South Wales 2109, Australia; Thermo Fisher Isotope Development Hub, Department of Earth and Planetary Sciences, Macquarie University, Sydney, New South Wales 2109, Australia; Department of Biomedical Research, Macquarie University, Sydney, New South Wales 2109, Australia; Université de Paris, Institut de Physique du Globe de Paris, CNRS, 75238 Paris, France

**Keywords:** Alzheimer's disease, brain potassium, isotope geochemistry, isotope metallomics, neurodegeneration, porcine model

## Abstract

Natural stable metal isotopes have shown utility in differentiation between healthy and diseased brain states (e.g. Alzheimer's disease, AD). While the AD brain accumulates some metals, it purges others, namely K (accompanied by increased serum K, suggesting brain–blood transferal). Here, K isotope compositions of Göttingen minipig brain regions for two AD models at midlife are reported. Results indicate heavy K isotope enrichment where amyloid beta (Aβ) accumulation is observed, and this enrichment correlates with relative K depletion. These results suggest preferential efflux of isotopically light K+ from the brain, a linkage between brain K concentrations and isotope compositions, and linkage to Aβ (previously shown to purge cellular brain K+). Brain K isotope compositions differ from that for serum and brain K is much more abundant than in serum, suggesting that changes in brain K may transfer a measurable K isotope excursion to serum, thereby generating an early AD biomarker.

## Graphical Abstract

**Graphical Abstract:**
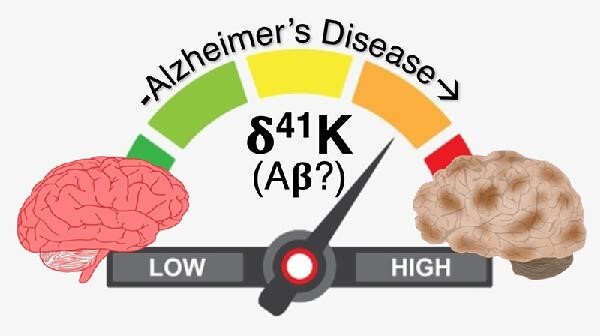
Potassium isotope compositions of minipig brain regions indicate heavy K isotope enrichment coincident with amyloid beta accumulation, suggesting a mechanistic linkage and potential for downstream signal transferal to blood serum (i.e. noninvasive diagnostic potential).

## Introduction

The role of biologically relevant metals—biometals—as well as their association with, and potential diagnostic value for, neurodegenerative diseases (like Alzheimer's disease, or AD), has led to increasing exploration and scrutiny of their systematics in biological systems.^1–3^ This premise has expanded into a burgeoning and promising field—*isotope metallomics*—which employs techniques conventionally used in geochemistry and analytical chemistry to constrain the abundance and distribution of biometals and their isotopes in biological systems.^4–16^ In general, the driving force for such research is to better understand metal homeostasis in the body and the underlying mechanisms of disease, and to potentially develop disease diagnostics that are more sensitive to the onset of a disease and can complement or predate conventional methods. With specific focus on biometals in relation to neurodegenerative diseases, it has been observed that metals such as Ca, Fe, Cu, and Zn accumulate in the brain as a function of age and/or the development of neurodegenerative disorders such as AD, where most metals are linked to the aggregation of amyloid β (Aβ) fibrils and the development of so-called senile plaques.^1,17–41^ While many such studies have focused on the earlier-mentioned linkages between metal accumulations and AD, complementary research has indicated that the AD-afflicted brain can also express metal deficits, namely for K (and Rb).^3^

The inherent connection between brain metal accumulation and neurodegeneration has led to the investigation of changes in total metal levels in the bloodstream as potential diagnostic metrics for AD, on the premise that disrupted metal homeostasis in the brain ought to manifest a correlative change in the abundance(s) of these metals in downstream bodily reservoirs that are more readily accessible for pathological study (and therefore diagnostics). However, because blood elemental abundances are, to varying degrees, subject to myriad conflating exogenous and endogenous factors such as sample size, sample processing, environmental factors, homeostatic transport mechanisms, genetics, cultural differences (e.g. diet), etc., the use of blood metal abundances alone as indicators of disease can be ambiguous and at times contradictory; see Cuajungco et al.^23^ and Acevedo et al.^41^ and references therein.

Exploring the utility of metal isotopes in medical research is a relatively new endeavor, yet this field has already shown considerable promise in understanding underlying mechanisms of disease and in developing disease biomarkers.^6,15,16,42–48^ The utility of metal isotopes in biological systems owes largely to the fact that isotopes generally fractionate due to: (i) changes in their bonding environments during exchange reactions (equilibrium isotope fractionation, e.g. healthy vs. diseased cells), where stronger bonds favor heavier isotopes^12,13^; and/or (ii) (nonequilibrium) kinetic effects during dominantly unidirectional processes such as diffusion.^49–51^ What is important in terms of the scientific value—and predictive power—of isotope metallomics is that both equilibrium and nonequilibrium isotope fractionation can be well described and modelled through *ab initio* theoretical calculations,^52–55^ allowing for mechanistic interpretations of empirical data.^15^ In brief, for equilibrium isotope fractionation, the isotopic composition of a given bodily reservoir, especially in relation to others (e.g. blood relative to brain tissue), is beholden to bonding environment; for kinetic isotope fractionation, along a chain of chemical reactions (e.g. unidirectional transport), lighter isotopes will tend to become enriched in reaction products.^54,55^ In neurodegenerative diseases, observations indicate that metals such as Ca, Fe, Cu, and Zn accumulates in the brain, and these metals are hypothesized to play a central mechanistic role in the development of AD; for such reason, most elemental and isotopic studies to date have focused on these metals,^25,27,28,56^ and in this context Cu and Zn have shown promise for utility in AD diagnostics.^12,13,57^ Importantly, where available, results from *ab initio* calculations agree with the magnitude and direction of isotope fractionation, even considering that such calculations simplify the bonding environment to that of amino acids that approximate the larger protein binding sites.^58^ For example, Cu isotope compositions in superoxide dismutase and metallothionein from postmortem human frontal cortex samples agree well with such theoretical calculations.^59^

However, observations for other metals—namely K (and Rb)—show significant decreases in the brain with AD, concomitant with an increased concentration in blood serum for K,^3^ indicating a linkage between the two and thus highlighting the potential for developing a noninvasive (i.e. blood pathology) AD biomarker based on K and its isotopes. In Roberts et al.,^3^ total K concentrations in human AD brain homogenates decreased by greater than 20% on average (24.4% decrease from ∼2 mg/g in control group), with an average concomitant increase in serum K of nearly 3% (2.6% increase from ∼145 mg/L). Potassium is essential to all known living organisms and is integral to a great many cellular and electrical functions within the body, with ∼98% of bodily K found in intracellular compartments.^60^ As the most abundant intracellular cation, K is pivotal in cardiac activity, membrane transport, neuromuscular functions and nerve impulse transmission; moreover, observations have shown that K is thus critical to diseases bound to these functions, e.g. hypertension, chronic kidney disease and neurodegenerative disorders such as AD.^61,62^

Specific to AD, major diagnostic pathologies are the formation of neurofibrillary tangles (caused by abnormal hyperphosphorylation of tau) and of senile plaques by extracellular deposition of Aβ fibrils, where in the latter case the soluble Aβ pool is indicative of disease severity.^63^ Increased K intake has been linked to reduced risk of dementia (especially vascular) in humans,^64^ and to reduced oxidative stress in an APP/PS1 murine model for AD (wherein increased K intake was also mechanistically linked to Aβ aggregation pattern and reduced tau phosphorylation).^65^ Related work on the association between Aβ and K in humans has determined a linkage between low K intake at midlife and low Aβ_42_ in cerebrospinal fluid (CSF) in late life (low CSF Aβ_42_ being a consistent indicator of AD and other forms of dementia); results suggest a biological and/or pathological link between K and AD in the precursor phase of AD.^66^ While the work of Mielke et al.^66^ awaits confirmation through replicate studies, the notion that K systematics early(ier) in life are related to AD/dementia risk later in life is further supported by separate research linking increased serum K to mild cognitive impairment in study cohorts (two) with average ages (∼60 yr) younger than that of typical AD diagnosis (>65 yr),^67^ especially when this finding is coupled to the observation that decreased K in the AD brain correlates with increased K in blood serum.^3^

All of the earlier-mentioned text strongly point to a connection between K and AD, and furthermore points to this connection being established during the precursor phases of the disease, which may occur decades before diagnosis using current methodologies, as highlighted by research on AD-related change points in CSF biomarkers^68^ (noting the specific biomarkers used therein were t-tau and Aβ_42_, both linked to K as previously described). K and its isotopes may be equally tenable as tools for understanding and/or diagnosing AD through biomarker development. This is especially true given the observed signal transferal to the bloodstream with respect to K concentrations.^3^ Furthermore, and while not the focus herein, the near omnipresence of K in terms of function within the body, where approximately 98% is intracellular,^60^ raises its potential as a disease diagnostic beyond AD to include other disorders in which intracellular K effluxes into the extracellular space and possibly into the bloodstream.^69,70^ Specific to K isotopes, this may be especially true when the K isotope composition of the intercellular compartment(s) is significantly different from that in blood.^71^

The study of the K stable isotope distributions in mammals is in its infancy, as the analytical challenges inherent to K stable isotope ratios measurements have only been operationally overcome in recent years.^71,72^ The recent advent of innovative analytical methods such as new generation collision-cell multi-collector inductively coupled-plasma mass spectrometers (CC–MC–ICP–MS) have opened new research avenues in the study of natural variations of K stable isotope ratios^71,73–75^ (Tacail *et al.*, in preparation). So far, only a few studies have explored biological systems, mainly in vegetal organisms.^76,77^ To date, the only published data for K isotopes in blood fractions are in Moynier et al.^75^ and Hobin et al.^78^ (Note: preprint data also exist in Higgins et al.^79^) Therefore, the potential of K stable isotopes in the study of mammalian (e.g. human) health and disease remains to be fully assessed, and this can only be done through targeted research. As with much isotope metallomics research, such investigations will be necessarily cumulative due to logistical, ethical and/or practical restrictions on sample availability and analyses.

In practical terms, K concentrations in most bodily reservoirs (e.g. plasma, organs, brain) are two or more orders of magnitude higher than that of transition metals (e.g. Fe, Cu, Zn) (e.g. Albarede et al.^43^ for human serum; Mahan et al.^14^ for porcine organs and blood fractions). This means that the determination of K isotope compositions in such reservoirs is more accessible, as significantly less sample is needed to generate statistically robust isotopic measurements. Moreover, because typical K concentrations in the brain are generally over an order of magnitude higher than that in blood plasma (e.g. 3000 ppm compared to 100–200 ppm, respectively),^14,43,80^ the isotopic signal of K disruption in the brain (especially that which may purge K into extracellular space, like apoptosis) is likely to be transferred to the bloodstream, a notion supported by previous observations for K concentrations in human brain tissue and serum.^3^ Taken together, these observations underscore an imperative for fundamental research aimed at characterizing the K isotope composition of the brain, other organs and blood, and more pointedly its investigation as a potential blood diagnostic for AD. [It is noted again that while the focus herein is on the possible utility of K isotopes in AD research, the potential utility of K isotope extends also to research of cardiovascular and organ diseases (e.g. chronic kidney disease).^69^]

The current study serves as the third instalment of an ongoing investigation aimed at building a foundational framework for research exploring the longitudinal evolution of biometals and their isotopes in the organs—and more specifically the brain—of porcine AD models (Göttingen minipigs). Mahan et al. (2018)^14^ investigated the homeostatic distribution of biometals and Zn isotopes in the organs and brain regions of the Göttingen minipig, and Mahan et al. (2020)^81^ focused on longitudinal changes in brain biometal concentrations and characterizing the Ca isotope composition of brain regions in two older animals, one single- and one double-transgenic, the same two animals further investigated herein. Experimental constraints relating to developing and maintaining large AD animal models dictate small cohorts of only one animal each of the two AD models explored, and a limited number of control (“wild-type”) animals. Therefore, it is important to note that both animals in Mahan et al.^82^ and herein represent a critical point in ageing and in the potential preclinical lifecycle of AD, and both are female (thus mitigating any potential but currently unknown effects of sex). Both animal ages are at the rough equivalent of human midlife, and although both animals had a predisposition for the development of AD, conventional imaging techniques (positron emission tomography, or PET) were not conclusive in determining the presence of senile plaques from insoluble Aβ fibril aggregation. The double-transgenic model expresses *in vitro* Aβ accumulation whereas the single-transgenic model does not, thus indicating that for the purposes of the present study (i.e. with respect to Aβ-related pathologies for AD), the single-transgenic model may act as an approximate control.

In the current work, the same AD single- and double-transgenic porcine models as in Mahan et al.^81^ have been further investigated to determine K isotope compositions of brain regions at midlife (where AD precursor changes are likely to start). Brain regions characterized include the amygdala (AM), basal ganglia (BG), brain stem (BM), cerebellum (CB), cerebral cortex (CX), and hippocampus (HC). The single-transgenic PS1 model (“M9”) was aged 70 months, or approximately 40 yr in human equivalency (∼6–7 ×ageing factor) and was genetically modified to express the human Presenilin 1 gene with the AD-causing M146I mutation (hereafter simply PS1).^82,83^ The double-transgenic APP/PS1 model (“M10”) was aged 43 months, or approximately 25 yr in human equivalency, and was genetically modified to express both PS1 and the human Aβ precursor protein gene with the AD-causing K670N/M171L double-mutation (“Swedish mutation,” hereafter simply APP).^83^ All other details of the porcine models can be found in Jakobsen et al.,^82,83^ and details of the elemental (Mg, P, K, Ca, Fe, Cu, and Zn) and isotopic (Zn and Ca) composition of their tissues and blood fractions can be found in Mahan et al.^14,81^

Small animal cohorts are necessary project constraints due to the exorbitant time and cost of developing transgenic porcine AD models and caring for the animals over protracted timeframes relative to, e.g. murine models. The current work builds upon (and incorporates) a comparatively large dataset to provide further context for continued research based on small cohorts of only one to two animals at different life stages. The dataset provided herein, while limited by these external considerations, details an important first look at K isotope systematics in the brain tissues of AD model animals at midlife and before clinical AD pathologies have been confirmed. Results from this work are used to speculate the viability of K isotopes as early biomarkers in AD, wherein observations strongly indicate great promise in this respect and a need for much future research.

## Methods

### Sample collection and digestion

Ellegaard Göttingen minipigs were raised, housed and euthanized according to Danish law on genetically modified animals, and experiments were conducted according to the Danish Animal Experiments Inspectorate (license no. 2006-561/1156; 2009-561/1733; 2017-15-0201-01251). All animals were raised under controlled laboratory conditions at Aarhus University, Denmark, and kept on the same diet from birth (elemental contents in Mahan et al.^14,81^). Brain tissue samples were recovered and dissected immediately after euthanization, and dissected samples were immediately snap frozen in liquid nitrogen and placed in polypropylene vials (Corning). Samples were kept at −135°C in temperature-controlled freezers at Aarhus University, Denmark, until transport in dry ice containers to the Institut de Physique du Globe de Paris (IPGP), where samples were again stored in temperature-controlled freezers until acid dissolution and further processing. Samples from two Göttingen minipig model types were included in this study, a single-transgenic PS1 model and a double-transgenic APP/PS1 model. The single- and double-transgenic animals in the current work tested negative for AD senile plaques *via* PET scan, but immunohistochemical analyses detected intraneuronal accumulation of Aβ_42_ in the brains from 10-month-old APP/PS1 (double-transgenic) pigs, which was not seen in age-matched wild-type pigs.^83^ Such accumulation may represent an early event in the pathogenesis of AD, and the transgenic models are therefore considered genetically predisposed to AD. Further developmental, genetic and medical details for both model types can be found in Mahan et al.^14^ and Jakobsen et al.,^82,83^ and further biometal, Zn and Ca isotope details can be found in Mahan et al.^14,82^

Large sample masses up to 1 g (except for small brain regions; typically 0.25 to 0.50 g) were dissected and digested to ensure representative and homogeneous sampling, as this was considered adequate for K given its more homogenized distribution in the brain relative to many other metals (see Mahan et al.^81^ for further details on sample masses; see, e.g. Becker et al.^87^ and Sussulini et al.^88^ regarding general brain K distribution). All samples were digested in a 1:10 mixture of hydrogen peroxide and concentrated nitric acid (HNO_3_, ∼70%) in clean polyfluoroalkyl vessels for 1 to 5 days (until solutions were translucent, indicative of total digestion) in the isotope geochemistry clean laboratory at IPGP.

### Potassium separation chemistry and isotopic analysis

Briefly, the K from biological samples and International Association for the Physical Sciences of the Oceans (IAPSO) seawater standard were purified following a 2-step cation exchange chromatography protocol using Biorad AG 50 W X-12 resin. This chemical purification procedure allows for a near full recovery of K (>99.5%) and contributes less than 20 ng K blank. Such a contribution is negligible in comparison with the typical sample size (>20 µg K) and is not expected to alter accuracy of δ^41/39^K—subsequently referred to as δ^41^K—measurement beyond analytical precision (Table 1).

**Table 1.** IAPSO D11-X12a seawater standard literature comparison

References	δ^41^K_SRM3141a_ (‰)	2δ	95% CI	*n*
Chen et al., 2021 (Chem. Geol.)^74^	0.12	0.06	0.02	8
	0.09	0.05	0.02	6
	0.10	0.07	0.03	6
	0.11	0.04	0.02	5
Hu *et al.*, 2018 (*Chem. Geol.*)^84^	0.14	0.04	0.01	11
	0.14	0.08	0.02	12
	0.13	0.07	0.03	7
Sun *et al.*, 2020 (GCA)^85^	0.14	0.07	0.03	7
Xu *et al.*, 2019 (*Chem. Geol.*)^86^	0.14	0.03	0.01	30
Average	0.12	0.04		9
Current study	0.12	0.04	0.04	7

The K purification and stable isotope measurements were carried out at the University of Bristol using the Proteus prototype instrument developed by Thermo Fisher Scientific (Bremen) in collaboration with the Bristol Isotope Group (University of Bristol, Bristol, UK). Proteus is a unique tribrid mass spectrometer with a pre-cell mass-filter (collectively, CC–MC–ICP–MS/MS). Instrument settings were tuned to optimize instrumental mass bias for stability, maintaining transmission and stability of the K signal intensity and thus the precision of δ^41^K measurements. The Ar^+^ and ArH^+^ species were neutralized using H_2_ as a reaction gas, carried by He for collisional focusing. Isotope ratios were measured using conventional standard-sample bracketing method (SSB). The NIST SRM-3141a reference solution was used as bracketing standard. The K stable isotope compositions are expressed in per mil, ‰, using the delta notation defined as follows:

\begin{equation*}
\delta {}_{}^{41}{K}_{{\mathrm{SRM}}3141a} = \ \left( {\frac{{{}_{}^{41}K/{}_{}^{39}{K}_{{\mathrm{sample}}}}}{{{}_{}^{41}K/{}_{}^{39}{K}_{{\mathrm{SRM}}3141a}}} - 1} \right)\end{equation*}

where ^41^K/^39^ K refers to the measured abundance ratios. An IAPSO seawater standard aliquot containing ca. 40 µg K was purified and analysed as a sample and used to assess the precision and accuracy of the method. Further details of the analytical method are part of an upcoming publication (Tacail *et al.*, in preparation).

In isotope chemistry, isotopic abundances in samples are typically discussed in reference to internationally recognized standards. Within this relativistic framework, a sample that is enriched in the light(er) isotopes of an element relative to a standard or other sample is referred to as “lighter,” and *vice versa*, a sample enriched in the heavy(ier) isotopes of an element is referred to as “heavier.” When per mil values (‰)—e.g. δ^41^K (‰)—are discussed (Equation 1), the terms lower (relatively light isotope enriched) and higher (relatively heavy isotope enriched) may also be used.

## Results

Biological reference materials are not widespread or readily available for K isotope analyses, and therefore IAPSO seawater was used as internal reference materials (Table 1). (Note: Moynier et al.^71^ and Hobin et al.^78^ detail K isotope compositions for widely available biological standards; however, these data were not published at the time of data acquisition for the current work.) The results for IAPSO seawater, with an average δ^41^K of 0.12 ± 0.04‰ (95% C.I. of the one sample *t*-test, *n* = 7 replicate analyses) agree with published values (averaging at 0.12 ± 0.04‰, 2SD, *n* = 9 literature values, see Table 1), indicating no measurable K isotopic fractionation during K chemical purification and/or analysis which is not accounted for by the SSB technique. In general, analytical resolution (two times the standard deviation, 2σ) for K isotope measurements within the current work was 0.09‰ or better (Table 2).

**Table 2.** K isotope data (Proteus CC–MC–ICP–MS/MS)

Sample ID^a^	Brain region	δ^41^K_SRM3141a_ (‰)	2 δ	95% ci	*n*
M9 (#5642 PS1 model, female aged 70 months)	AM	−0.39	0.03	0.04	3
	BG	−0.59	0.04	0.05	3
	BM	−0.70	0.05	0.03	5
	CB	−0.80	0.05	0.03	6
	CX	−0.67	0.09	0.05	6
	HC	−0.50	0.08	0.10	3
M10 (#6170 APP/PS1 model, female aged 43 months)	AM	−0.40	0.09	0.07	4
	BG	−0.39	0.10	0.08	4
	BM	−0.41	0.10	0.05	6
	CB	−0.60	0.03	0.04	3
	CX	−0.48	0.04	0.05	3
	HC	−0.34	0.04	0.19	2
IAPSO D11-X12a		0.12	0.04		7

^a^Sample IDs and all further data and porcine model information can be found in Mahan et al.,^14,81^ and references therein. AM—amygdala, BG—basal ganglia, BM—brainstem, CB—cerebellum, CX—cerebral cortex, and HC—hippocampus.

**Fig. 1:**
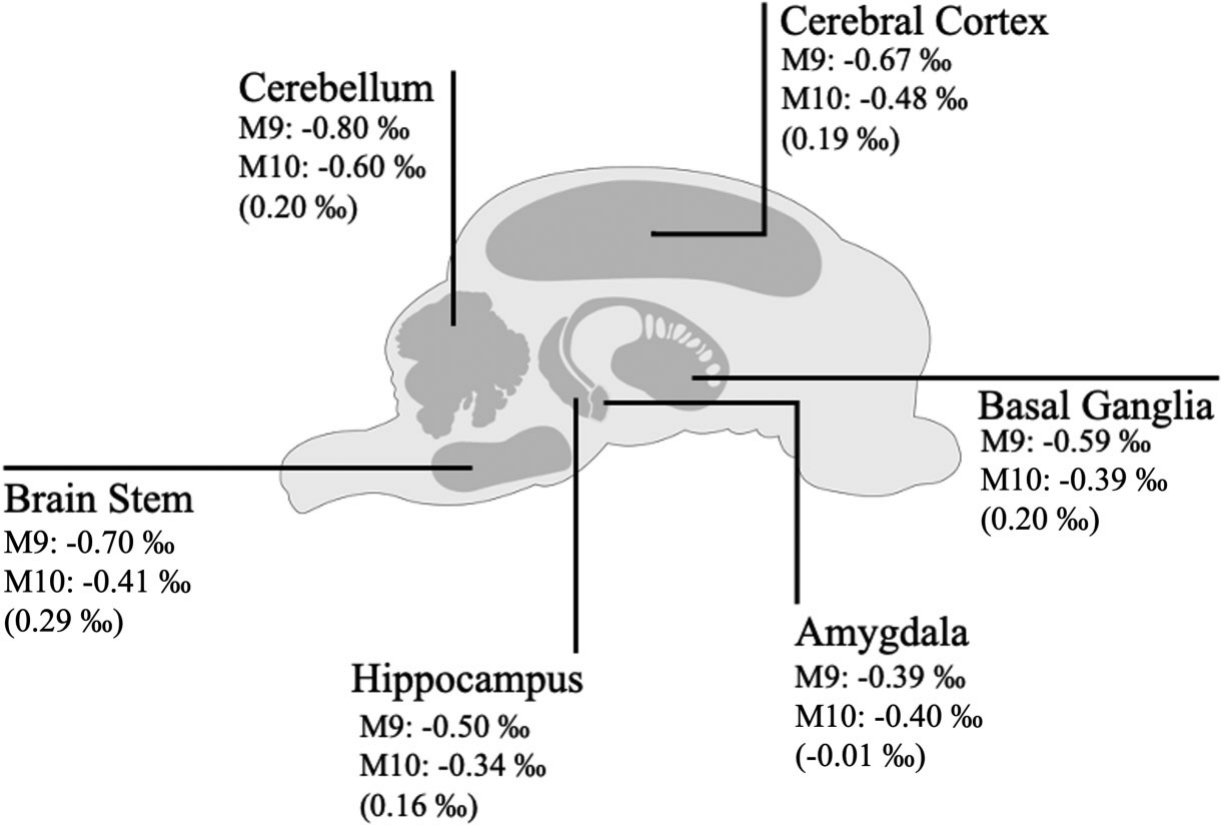
Anatomical schematic of the Göttingen minipig brain with K isotope ratios—δ^41^K—for each region. Values in parenthesis are calculated heavy K isotope enrichment in porcine model M10 (APP/PS1) relative to M9 (PS1 only) (i.e. δ^41^K offset between APP/PS1 and PS1 animals, respectively). See Table 1 and Fig. 2 for associated analytical uncertainties.

**Fig. 2:**
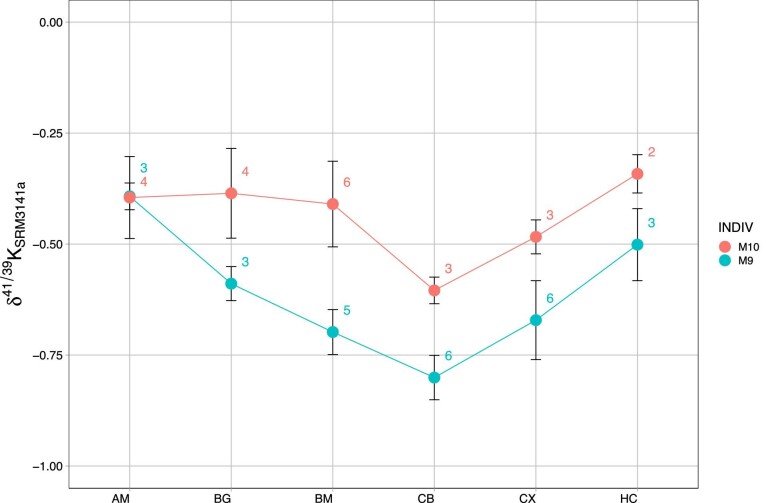
Spider plot of K isotope results highlighting the general ∼0.20‰ increase in δ^41^K for the younger double-transgenic animal (M10) relative to the older single-transgenic animal (M9) (except for the amygdala). AM—amygdala, BG—basal ganglia, BM—brainstem, CB—cerebellum, CX—cerebral cortex, and HC—hippocampus. Numbers next to data points indicate number of replicate analyses; analytical uncertainties represented by 2σ ± brackets for each sample (determined from replicate analyses).

**Fig. 3:**
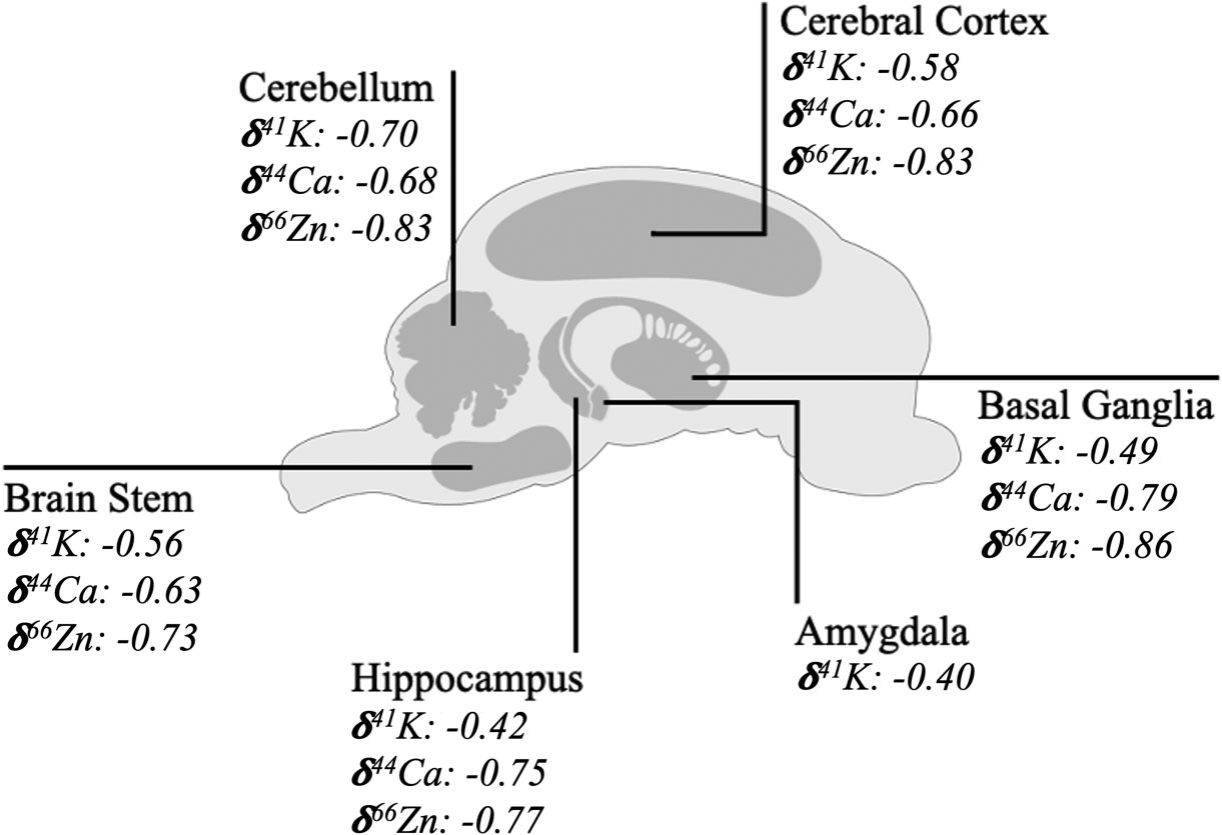
Collated isotopic data to date for minipig brain regions from this work and Mahan et al.^14^ and Higgins et al.^79^ (Note: data for δ^41^K are indicative only, as isotopic composition varies considerably both within and between animal models.)

Potassium isotope compositions, δ^41^K, for Göttingen minipig brain regions within the current work ranged from −0.80 to −0.34‰ (Table 2, Figs. 1 and 2), yielding a variability between different brain regions of ∼0.5‰. Such dynamic range in K isotope composition is considerable relative to all previous K isotope studies (e.g. Wang et al.^89^ and references therein), and is particularly large within the context of biological systems, as all other metal isotope systems to date have exhibited a more-or-less constant isotope composition across brain regions.^14,81^ Keeping in mind the variability observed in K isotope compositions and small *n* (and therefore indicative nature of averaging), averages for each brain region were calculated and combined with other metal isotope systems investigated in Göttingen minipig brain regions to date (Ca and Zn) in order to collate this information for reference (Fig. 3); in general, the brain is marked by isotopically light compositions for metals, indicative of a bonding environment that preferentially incorporates the light isotopes of K, Ca, and Zn.

Potassium isotope compositions across brain regions in both animals nearly parallel one another, except for the amygdala where values are statistically identical (Fig. 2). The K isotope composition of brain regions in the (younger) double-transgenic model (M10) is on average heavier by ∼0.20‰, except for the brainstem where it is ∼0.30‰ heavier and the amygdala where the K isotope composition for both animals is statistically identical (Table 2, Figs. 1 and 2).

**Fig. 4:**
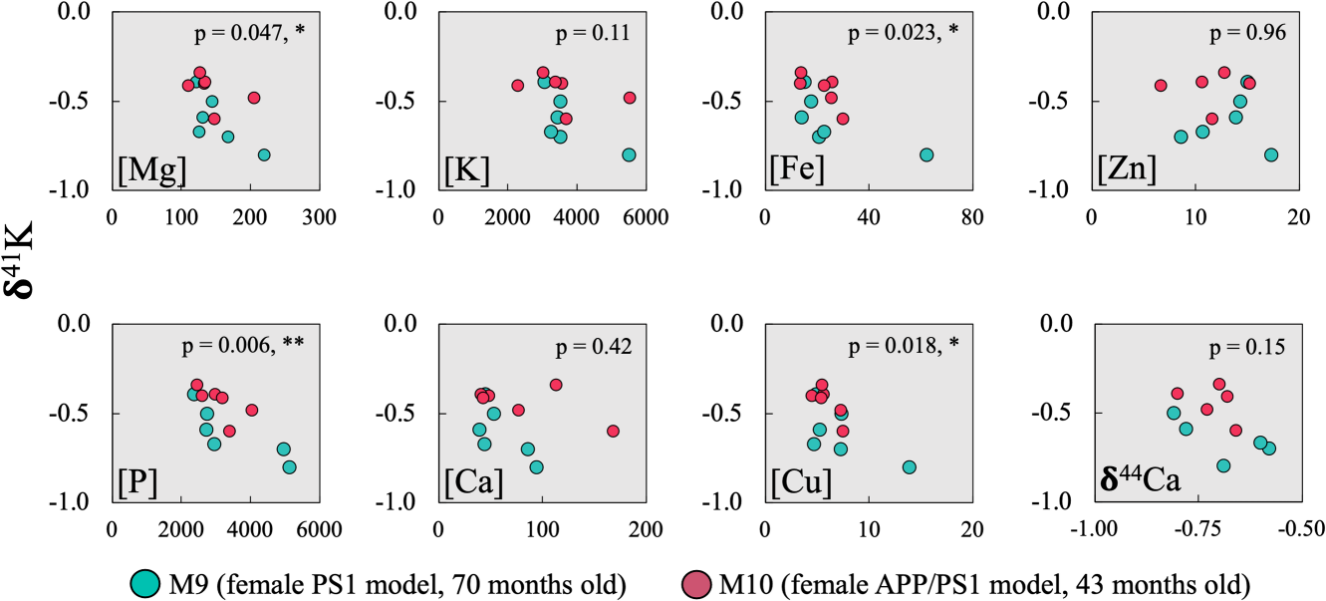
Plots of δ^41^K for the single- and double-transgenic AD model animals in the current work as a function of concentration for biologically relevant elements (biometals and P) and δ^44^Ca (color coded as mentioned earlier). Reproducibility of measurements via Q–ICP–MS was better than 5% (RSD) for all elements,^79^ and symbols encompass analytical error; analytical uncertainties for K isotope measurements in Table 2. Statistically significant relations were determined for Mg(*), P(**), Fe(*), and Cu(*). The increasing power of the statistical significance is defined as: *P* ≤ 0.05 (*); *P* ≤ 0.01, **; *P* ≤ 0.001, ***; and *P* ≤ 0.0001, ****.

**Fig. 5:**
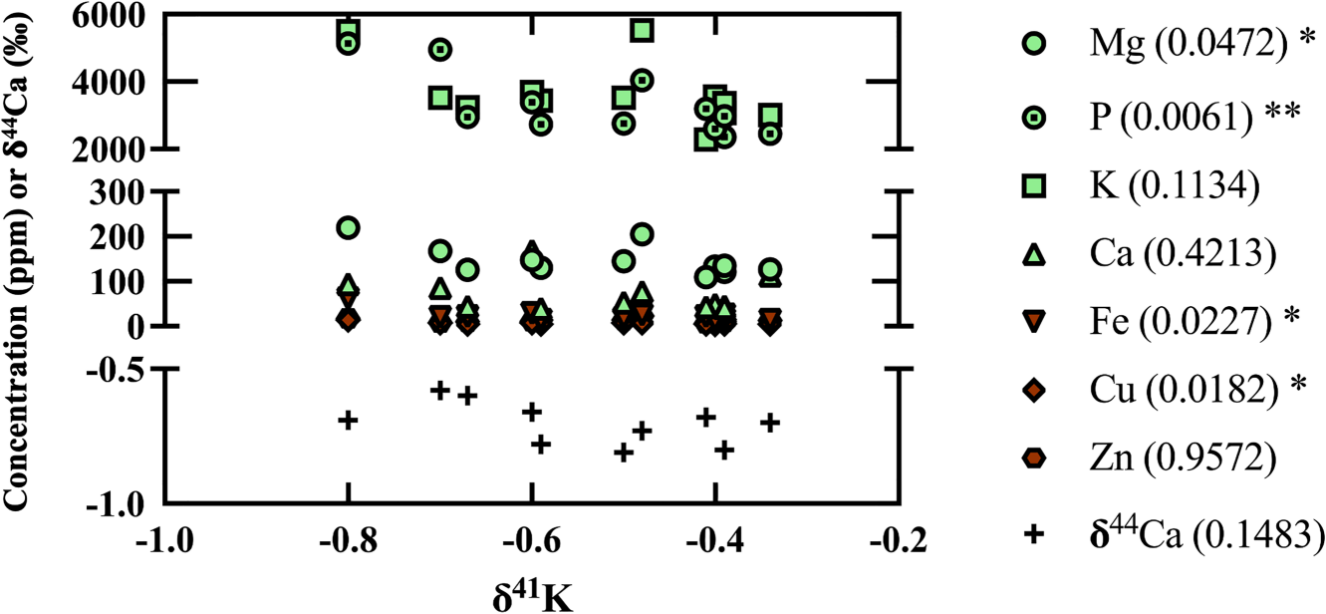
Statistical results for dependence of metal concentrations and δ^44^Ca as a function of δ^41^K (unpaired *t*-tests in GraphPad Prism with 2-tailed *P-*values).

Like that previously done in Mahan et al.,^81^ and again noting the inherently limited *n* for the dataset, δ^41^K for both AD model animals were compared to contents of biologically relevant elements (and δ^44^Ca) (Fig. 4), and statistical analyses performed (e.g. unpaired *t*-tests in GraphPad Prism for δ^41^K vs. other analytes; Fig. 5), to discern any potential linkages and their statistical relevance. Determination of the Pearson correlation coefficient (Pearson's *R*) for δ^41^K vs. AD model type, with *r* = 0.61 and *P* = 0.036(*), confirms the statistical significance of the trend toward heavy isotope enrichment in the double-transgenic animal. Regarding other analyte trends, statistically significant trends were determined for Mg(*), P(**), Fe(*), and Cu(*) (Fig. 5). No statistically significant trend was found between δ^41^K and K concentration, while Mahan et al.^81^ found a statistically significant increase in K with age (*P* = 0.0076, **); however, despite this more general trend in the larger cohort, and with the exception of the cerebral cortex and amygdala (see “Discussion”), K concentrations in the double-transgenic animal are lower than in the much older single-transgenic animal.^81^

## Discussion

The current dataset is limited by experimental factors, and moreover by sparse K isotope data in biological materials for comparison, with that of Moynier et al.^71^ and Hobin et al.^78^ being the only published contemporary dataset for comparison (see also the early historical work of Mullins et al.^90^ and preprint of Higgins et al.^79^). With these caveats well noted, the data nevertheless provide insight into the K isotope systematics of the brain and how such data might be expanded upon and utilized in for future aimed at the development of disease biomarkers such as those for AD.

It bears repeating that the two animals explored herein and in Mahan et al.^81^ (both female, removing sex as a variable) represent a critical point in ageing and in the potential preclinical lifecycle of AD, as both are at the rough equivalent of human midlife. Both animals had a predisposition for the development of AD; PET was inconclusive in determining the presence of senile insoluble Aβ fibril aggregation; however, the double-transgenic model expresses *in vitro* Aβ accumulation. Taken together, this means that any observations from the current work and the larger cohort (Mahan et al.^14,81^) may aide in the evaluation and determination of preclinical manifestations of AD. These data detail the first K isotope measurements made in brain tissues of a large animal model, and the first to explore this in AD models. Of critical import, the present work builds on a much larger dataset toward the goal of an internally consistent, longitudinal assessment of metal and metal isotope viability/utility in AD diagnostics; the current work and larger cohort study is unique in currently representing the only isotope metallomics data and observations in large animal models of AD. While any interpretations are naturally restricted to speculation—due to observations from two animals, one of each AD model—the dataset and derived preliminary hypotheses are of great value to the community as this new horizon is explored. Prior to exploring implications and hypotheses derived from the current work, the statistically significant relationships within the data are parsed out and linked back to previous observations within the larger cohort and in the literature.

### Correlative relationships between δ^41^K, biologically relevant elements and δ^44^Ca

**Fig. 6:**
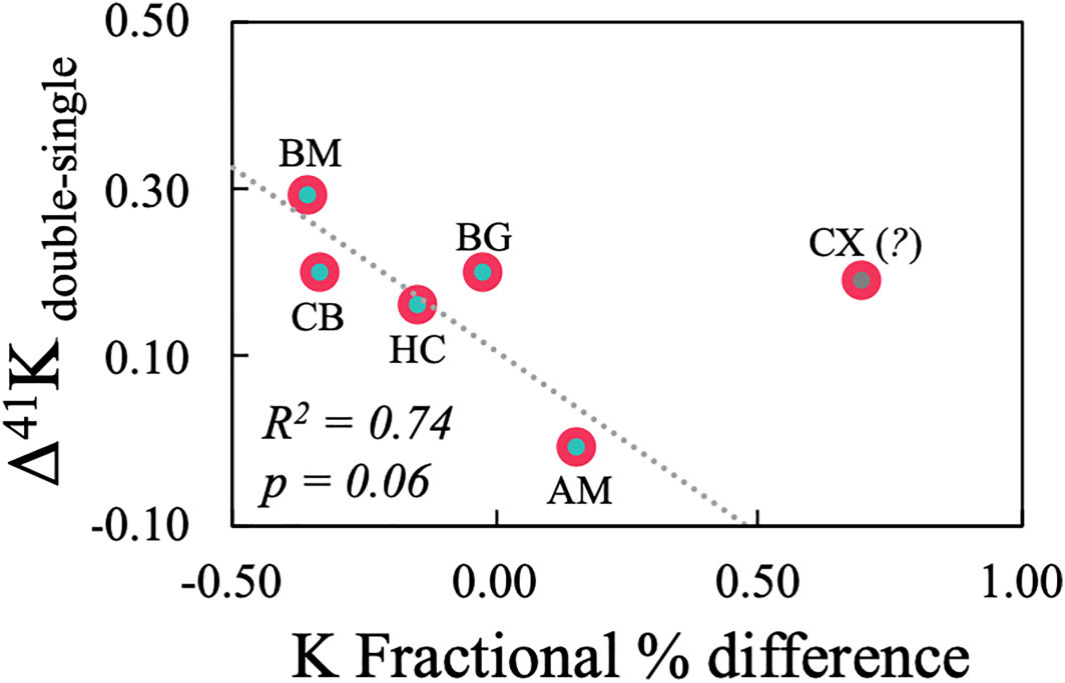
Heavy K isotope enrichment (high δ^41^K) in the double-transgenic model (M10) relative to the single-transgenic model (M9), expressed as Δ^41^K_double–single_ (δ^41^K_M10_–δ^41^K_M9_), as a function of the % difference in K concentration in the same reference frame (percentage depletion in M10 relative to M9). All brain regions fall on a discernible trendline (except for the cerebral cortex, CX), suggesting a mechanistic linkage between brain K depletion (associated with AD) and δ^41^K. At present, the reason for the excursion of the cerebral cortex off this trendline is unknown.

Previous observations in Mahan et al.^81^ determined statistically significant relationships between age and the biologically relevant elements Mg (*), K (**), Ca (*), Fe (****), and Cu (****), wherein metal concentrations increase with age. With respect to K, this may at face value seem at odds with observations in humans; however, such studies typically normalize for age to isolate the effects of AD,^3^ wherein brain K decreased as a function of the disease (with a concomitant and likely linked increase in serum K). In relation to the current work, while a significant age effect was determined for K(**) within the larger cohort to date, when focusing on only the two AD model animals herein, it is observed that K concentrations in all but two brain regions (cerebral cortex and amygdala) of the younger double-transgenic pig are lower than that in the older single-transgenic animal by up to 35%. The lack of a K decrease in the cerebral cortex of the double-transgenic animal cannot currently be constrained; however, the lack of K depletion in the amygdala (slight relative enrichment) is potentially explained by the fact that the amygdala is typically affected later in the development of AD (e.g. after brain stem and hippocampus), and its initial changes are not bilateral (i.e. affecting only the right amygdala first).^91^ When including the cerebral cortex, no trend can be discerned between δ^41^K and brain K depletion in the double-transgenic animal relative to the single-transgenic animal (*P* = 0.51). However, when excluding the cerebral cortex, a discernible and significant pattern emerges (*R*^2^ = 0.74; *P* = 0.06) (Fig. 6), indicating a linkage between AD, brain K concentrations and δ^41^K. An investigation of δ^41^K vs. other analytes for these two animals (Mahan et al.^81^ and current work), yielded significant correlations were found for Mg(*), P(**), Fe(*), and Cu(*) (Fig. 5). The significance of the relationship(s) between δ^41^K and Mg and P is currently unknown; however the relationship between Fe and Cu aligns with the accumulation of these metals in the brain as a function of AD progression (e.g. Bush^31^ and Mahan et al.^92^), further bolstering the notion that the covariations seen herein are manifestations of AD-related processes, namely those involving Aβ. While further investigation is certainly required, these observations beckon for further research with respect to K isotope systematics and AD.

### Potassium isotope systematics and speculative implications for an AD biomarker

The pronounced variability in δ^41^K among the brain regions contrasts with that seen for all other metal isotope systems investigated thus far, where isotope composition is largely constant throughout the brain Mahan et al.^14,81,92^ While it may be improbable that internal differences among brain regions could be discernible after downstream transport of K from the brain into the blood (e.g. determining an efflux from the amygdala vs. the cerebellum into the bloodstream), future work in postmortem brain tissue samples in animal models and humans alike is merited, as this variability indicates that the brain is a dynamic environment for K and its isotopes. Changes in the K concentration and isotope ratio as a function of disease may provide insight into underlying mechanisms, because changes in K bonding environment may elicit changes in K isotope compositions, thereby shedding light on mechanistic deviations in the brain, as suggested herein (Fig. 6). While the current data are inherently limited in terms of statistics and prescriptive/predictive power, preliminary hypotheses based on such data are still of great value and can be used to guide future research, ultimately validating or modifying/nullifying such hypotheses.

There are several possible pathways through which brain (and plasma/serum) δ^41^K could be altered with respect to AD. K is bound to aspartate and glutamate in Na/K-ATPase, the activity of which is thought to be altered in AD (leading to decreased brain K).^93^ No *ab initio* or experimental data exist for isotopic fractionation of K isotopes between binding sites in biological materials; however, the general rule that heavy isotopes will preferentially be found in stronger bonds would suggests that K in Na/K-ATPase should be isotopically heavier than extracellular free/hydrated K^+^, such as has been seen for other isotope systems like Zn.^10,58^ Therefore, altered Na/K-ATPase activity resulting in the loss of intracellular K should result in a decrease in brain δ^41^K (loss of heavy isotope enriched Na/K-ATPase). This would conceivably manifest a lower average δ^41^K (negative δ^41^K excursion) in the double-transgenic animal brain. This is the opposite of that observed (Fig. 1) and may suggest a minimal influence and/or overprinting of such a mechanism.

Alternatively, as mentioned, the double-transgenic model in the current work displayed intraneuronal Aβ_42_ accumulation and overexpression of Aβ*PPsw*,^83^ previously linked to high Ca concentrations across the brain regions in this animal (save the brain stem) and thought to be derived from the synergistic effect of APP and PS1 (see Mahan et al.^81^ and references therein). Separate *in vitro* work on mixed cortical cultures has shown that Aβ can induce K^+^ efflux out of the afflicted region.^3,94^ On the same general principle that K^+^ (“free” or hydrated K) ought to be isotopically light relative to cellularly bound K (e.g. bound to aspartate/glutamate), this would manifest an increase in brain δ^41^K—a positive δ^41^K excursion—for the double-transgenic animal (relative to single transgenic, which did not express Aβ accumulation). That is to say, the presence of Aβ accumulation should increase the value of brain δ^41^K, in line the typically higher (by ∼0.20‰) δ^41^K values as seen in the double-transgenic model that expressed brain Aβ accumulation. While tentative, this explains the generally heavier K isotope composition of brains regions in the double-transgenic minipig in the current study and the statistical significance of this relationship (Figs. 1 and 2; Pearson's *R* for δ^41^K vs. AD model, *P* = 0.036,*).

If indeed what is being tracked are positive δ^41^K excursions in the brain caused by AD-related precursor changes (e.g. soluble Aβ accumulation), then first-order inferences—again wholly tentative—can be made with regards to region-to-region δ^41^K systematics. The generally consistent positive offset of ∼0.20‰ in the double-transgenic brain—e.g. for the basal ganglia, cerebellum, cerebral cortex, and hippocampus—suggests that with respect to underlying mechanisms that may affect K isotope compositions, these brain regions are affected similarly both with respect to timing and spatial extent (magnitude) of change. The statistically identical amygdala K isotope compositions of both animals (and lack of depleted K concentration) may remark on the fact that the amygdala is affected later and nonbilaterally in the development of AD.^91^ Following a similar logic, the brainstem of the double-transgenic animal displays the largest positive δ^41^K excursion within this study (nearly 0.30‰; Fig. 2), and it is possible that the high(er) magnitude positive δ^41^K excursion seen in the brainstem of the double-transgenic animal is a temporal effect due to this structure of the brain being the first affected during AD at its earliest stages.^95^

If these hypotheses prove true, there is very strong impetus for future work investigating blood δ^41^K as a function of AD. The K isotope composition of the ERM-CE196 bovine blood certified reference material (CRM) has a distinctly high δ^41^K relative to all other tissue samples analysed for terrestrial mammals to date in Moynier et al.^71^ and the current work, and similar results were determined for the Seronorm (human) whole blood analysed in Hobin et al.^78^; additionally they^78^ analysed Seronorm (human) blood serum with a determined average δ^41^K of −0.30 ± 0.04‰, which is also resolvably higher than brain δ^41^K compositions determined herein. While still in the preprint phase (at time of writing) and not strictly comparable for δ^41^K due to normalization to diet/plasma, the work of^79^ also determined that in *R. norvegicus* (common brown rat), blood plasma has significantly higher δ^41^K relative to the brain (cerebrum average δ^41^K of −0.25 ± 0.15‰; cerebellum average δ^41^K of −0.38 ± 0.21‰, relative to plasma) (and red blood cell, RBC, average δ^41^K of 0.40 ± 0.08‰). Cumulatively, these observations—coupled with the fact that 98% of bodily K is bound in muscle, skin, subcutaneous tissue, etc.—suggests that any change in the body that results in the efflux of brain K+ into the bloodstream may impart a change in the K composition of the blood, most likely in the sera/plasma fraction,^69,70^ with the latter supported by the fact that signal transferal has previously been demonstrated for K concentrations in human AD subjects, wherein a decrease in brain K was associated with a measurable increase in serum K.^3^

With respect to isotope systematics, there is a lack of *ab initio* calculation for K isotopes to draw from. However, and while the two cannot be considered directly analogous, we can make first-order inferences by looking to Cu isotopes. Perhaps most relevant is the observation of a light Cu isotope excursion in human blood serum from ovarian cancer, as this provides a rough analogue with respect to physical reservoir size (noting that magnitude decrease in K from brain to serum is order of magnitude larger). In Toubhans et al.^96^ it was found that serum δ^65^Cu was significantly lower in ovarian cancer patients relative to controls, while tumor biopsies revealed higher δ^65^Cu values relative to adjacent healthy tissues, suggesting ^65^Cu sequestration in tumors and subsequent relative ^63^Cu enrichment in serum. While in such a scenario the relative re-apportioning of isotopes is the exact opposite to that hypothesized herein (light isotope efflux as opposed to heavy isotope uptake), in terms of mass balance the result is similar. Without *ab initio* calculations for possible K isotope binding sites, no further speculation or calculations can be made regarding mass balance and whether an efflux of isotopically light K from the brain would induce a change in serum that is measurable. However, from first principles and Fujii et al.^58^ it is reasonable to make the plausible assumption of heavy metal isotope enrichment when the metal is cellularly bound as opposed to “free” (a hydrated ion), and that the typical isotopic offset between the two is often larger than 0.5‰; this would suggest a similar scenario for K. While of course qualitative only, this cumulatively suggests that—at a minimum—there is sufficient likelihood of a measurable offset in serum δ^41^K between human AD and control sera to justify further considered interrogation of this hypothesis.

In brief summary, flushing of isotopically light K from the brain should drive brain δ^41^K higher (as seen herein), while driving blood sera/plasma δ^41^K lower. The likelihood of this process imparting a measurable change in blood sera/plasma δ^41^K is relatively high given that brain K concentrations are one or two orders of magnitude higher than that in blood plasma (and similar is true for other organs). [Note: published data do not permit an understanding of potential differences in K isotope compositions due to K contamination in the CRMs, e.g. from the possible use of K-based EDTA anti-coagulants (e.g. no information given in ERM-CE196, ERM Certificate 2013), and thus parts of the earlier-mentioned discussion await future work to clarify.]

## Concluding Remarks

In comparison to the data of Moynier et al.^71^ and Hobin et al.,^78^ the K isotope compositions of brain regions determined in the current work are well within the range of values reported therein for land mammals (approx. range in δ^41^K of −1.0 to 0.1‰). More pointedly, K isotope data in the current work lie within the range of all largely cellular materials (e.g. muscle and organ tissue), indicating *a priori* that organ/tissue samples for land mammals likely fall within the same range, and this generally framework thus likely extends to humans.

A change in K isotope composition in a downstream reservoir (e.g. blood sera/plasma) requires that the K isotope composition of the effluxing reservoir (e.g. an intracellular brain compartment) be different from that of the reservoir that it is flushing into (i.e. the bloodstream), and that the effluxing mass is large enough to measurably influence the isotopic composition of the reservoir that it is flushing into (akin to what has been observed for Ca isotopes in osteoporosis.^16^) The appreciably different K isotope composition of brain regions as determined herein (relative to published data for blood fractions) indicate that significant loss of brain K—as occurs with AD—may impart a measurable change in the K isotope composition of blood, especially in the plasma/sera, thereby acting as a biomarker (diagnostic indicator) of brain dyshomeostasis and AD. If such investigations proved fruitful, e.g. through observation of a linkage between AD (and its severity) and δ^41^K excursion in blood, this would compel future efforts in this general field, but also perhaps a targeted study leveraging quantification of brainstem atrophy (e.g. through volume-loss morphometry, see Ji et al.^95^) and blood δ^41^K perturbation, toward potentially combining the two as a noninvasive multi-factor AD diagnostic.

The present work (and that of Moynier et al.,^71^ Hobin et al.,^78^ and Higgins et al.^79^) provides a clear impetus for continued exploration of K isotopes in the brain, blood and other organs, and linkages between them all, to further constrain the underlying processes at hand, and to further elucidate the potential diagnostic power of K isotopes in disease. This applies not only to neurodegenerative diseases like AD, but to other diseases where K isotope systematics suggest manifestation of a blood (e.g. serum) δ^41^K excursion as a function of disease. For AD specifically, the current work provides a compelling case for future work dedicated to understanding the systematics of K isotopes in AD, its relationship to early biochemical and morphological changes associated with AD (e.g. Aβ accumulation and brain atrophy, respectively), and its ultimate diagnostic potential.

On a pragmatic note, due to significantly higher K concentrations in bodily reservoirs relative to many other metals investigated within isotope metallomics research (e.g. K sometimes 100–1000× higher in concentration), much less sample mass is required to perform reliable K isotope measurements. Moreover, current generations of MC–ICP–MS instrumentation (e.g. Nu Sapphire and Thermo Fisher Neoma) allow for more straightforward and routine analyses of K isotope ratios. This alleviates many of the procedural, technical and practical limitations imposed on other isotope systems, and mitigates the previous hindrances of K isotope analyses.

Finally, previous work in K systematics within the body have also detailed a number of other disorders which may induce purging of intracellular K into the bloodstream, such as kidney and/or colon disorders, and cardiovascular diseases.^70^ With respect to diseases of the kidney, with a K isotope composition akin to that of the brain (average δ^41^K of −0.34‰; from Moynier et al.^71^) a similar transmission of signal may be generated. There is potential that such a signal could be generated, e.g. in the progression of chronic kidney disease, where K excretion through the gut increases (Udensi and Tchounwou^70^ and references therein).

All things considered, there is great impetus and need for continued and expansive research into K isotope systematics in biological systems, in AD and in other diseases.

## Data Availability

The data underlying this article are available in the article.
